# Systematic review: Development of a person‐centered care framework within the context of HIV treatment settings in sub‐Saharan Africa

**DOI:** 10.1111/tmi.13746

**Published:** 2022-04-01

**Authors:** Malia Duffy, Caitlin Madevu‐Matson, Jessica E. Posner, Hana Zwick, Melissa Sharer, Antonia M. Powell

**Affiliations:** ^1^ International Division John Snow, Inc. Boston Massachusetts USA; ^2^ Public Health Saint Ambrose University Davenport Iowa USA; ^3^ Global Health Institute Duke University Durham North Carolina USA

**Keywords:** client‐centred care, HIV, HIV care continuum, HIV treatment, person‐centred care, sub‐Saharan Africa

## Abstract

**Objectives:**

Person‐centred care (PCC) meets the needs of individuals by increasing convenience, providing supportive and culturally appropriate services to diverse populations, and engaging families, communities, and stakeholders in planning and provision of care. While the evidence demonstrates that PCC approaches can lead to clinical improvements across the HIV care continuum, it is not yet well defined in the context of HIV service delivery.

**Methods:**

A systematic review was conducted to define PCC practices for HIV treatment services in health facilities in sub‐Saharan Africa. Data synthesis led to the development of a PCC framework including domain and sub‐domain development. The study team used the Effective Public Health Project Practice tool for quantitative studies to assess the quality of the included studies.

**Results:**

Thirty‐one studies from 12 countries met the inclusion criteria, including 56,586 study participants (females 42%–100% and males 0%—58%), resulting in three major domains and 11 sub‐domains. These include staffing (sub‐domains of composition, availability, and competency); service delivery standards (sub‐domains of client feedback mechanisms; service efficiency and integration; convenience and access; and digital health worker support tools); and direct client support services (sub‐domains of psychosocial services, logistics support, client‐agency, and digital client support tools). Twenty‐five of the person‐centred interventions within these domains resulted in improvements in linkage to care, treatment retention, and/or viral suppression.

**Conclusions:**

The PCC framework can help to provide a more consistent classification of HIV treatment interventions and will support improved assessment of these interventions to ensure that people receive personalised care.

## INTRODUCTION

Sub‐Saharan Africa (SSA) is home to 12% of the world's population and 71% of the global HIV burden [1]. The region was not on track to reach UNAIDS 90‐90‐90 targets in 2018, as only 81% of people living with HIV (PLHIV) knew their status; 64% were on treatment, and 52% were virologically suppressed [2]. Since that time, the COVID‐19 pandemic disrupted HIV systems and service delivery and introduced additional barriers to achieving the UNAIDS Goals of ending AIDS by 2030 [3].

UNAIDS has three people‐centred 2025 targets: comprehensive HIV services; people‐centred and context‐specific HIV services; and removal of societal and legal impediments to an enabling environment for HIV services [3]. WHO also has a global strategy on people‐centred health services, noting benefits across all aspects of health care [4]. Person‐centred care (PCC) was also recently supported by a consensus statement from global HIV experts, emphasising the need for a more person‐centred HIV agenda while recognising the lack of a common understanding of the definition and core values of PCC [5]. The President's Emergency Plan for AIDS Relief (PEPFAR) defines PCC as meeting the needs of individuals by increasing convenience, making services supportive and accessible, providing friendly services to diverse populations, and engaging communities and stakeholders. PEPFAR increasingly prioritises PCC through differentiated service delivery approaches for antiretroviral therapy (ART), particularly multi‐month dispensing and community‐based ART dispensation [6]. In line with these efforts and to reduce risk during the COVID‐19 pandemic, innovative solutions further decongest health facilities, reduce travel burden, and make HIV treatment more convenient and responsive to diverse populations' needs [7].

Evidence suggests that person‐centred HIV treatment activities lead to improved health outcomes. Clients offered choices for ART collection demonstrated at least equal adherence, retention, and viral suppression (VS) outcomes as clients under a standard of care (SOC), and in some cases, significantly improved outcomes across the continuum while reducing gender disparity gaps [8, 9, 10]. Other activities characterised by PCC, such as offering adolescent‐friendly HIV services, have demonstrated improvements in treatment adherence [11, 12, 13, 14]. Community and workplace‐based and male‐led peer support groups have also shown improvements across the HIV care continuum [15].

While the evidence demonstrates that PCC approaches can lead to clinical improvements, PCC in the context of HIV treatment service delivery has not yet been well defined or measured in the literature. Responding to this gap, a team of researchers and program implementers from John Snow, Inc. (JSI) undertook a systematic review to define PCC characteristics within the context of HIV treatment provided by health facilities in SSA. To the best of our knowledge, this is the first systematic review that attempts to develop a definition and framework for PCC within the context of HIV treatment. Policy‐makers, program designers, and clinicians may use the findings to prioritise interventions aligned with PCC, contributing to the global goal of ending inequalities and the AIDS epidemic by 2030.

## METHODS

The study team (MD, CMM, JP, and HZ) followed the PRISMA standards of quality for reporting systematic reviews [16], developing a study protocol that outlined the systematic review process, identified study team member's roles, and clearly defined eligibility criteria for articles included in this study. This systematic review does not involve human subject research so was exempted by the JSI Institutional Review Board. The study team registered the systematic review on the International Prospective Register of Systematic Reviews (PROSPERO CRD42021246011).

### Search strategy and study collection

The study team conducted an electronic database search including PubMed and CINAHL using the MeSH and title and abstract terms related to the search words in Box 1. The [Link], [Link] section contains a detailed description of the search strategy. Two study team members independently reviewed titles and abstracts to determine inclusion eligibility. When there was disagreement, a third study team member made the final decision (interrater reliability 87%). Subsequently, two study team members independently reviewed full‐text articles of included abstracts to make final inclusion determinations. When there was disagreement, a third study team member made the final determination on inclusion (interrater reliability 84%).

BOX 1 **Search terms**

Person‐centered care OR client‐centered care AND HIV
Differentiated service delivery AND HIV
Adolescent‐friendly AND HIV treatment
Male‐friendly AND HIV treatment
Child‐friendly AND HIV treatment
Pregnant women‐friendly AND HIV treatment
Key population‐friendly AND HIV treatment
Men AND HIV treatment AND access OR engagement OR retention OR adherence OR uptake
Pregnant women AND HIV treatment AND access OR engagement OR retention OR adherence OR uptake
Women of childbearing age AND HIV treatment AND access OR engagement OR retention OR adherence OR Uptake
Adolescent AND HIV treatment AND access OR engagement OR retention OR adherence OR uptake

Child AND HIV treatment AND access OR engagement OR retention OR adherence OR uptake.

The study team used the PICOS framework (person, intervention, comparison, outcome, study design) to determine inclusion. Articles were included if they were in English and published between January 2016 and April 2021 to ensure they incorporated the most recent evidence base. Studies had to focus on clients accessing HIV treatment services or health care workers (HCW) providing services to PLHIV (person). Studies had to describe an HIV treatment program or identify/describe elements of PCC that contributed to HIV treatment uptake, retention, and/or VS in clinical settings. Community settings were excluded because the purpose of this review is to inform the development of a PCC tool for use at facilities (intervention). Studies could but were not required to compare different models of PCC (comparison). Studies were required to include health outcomes related to the PCC intervention to merit inclusion. Although PCC interventions are not necessarily designed to show clinical outcomes, the study team required outcome reporting to help understand whether the interventions led to positive, negative, or no changes, to examine their potential contribution within a PCC framework (outcome). Any study type other than reviews and study protocols were included (study design).

### Data extraction and synthesis

A realist synthesis framework was applied wherein the study team defined the scope of the review, examined the evidence base, and used a data extraction table that was pre‐tested and refined prior to use to provide a framework for synthesis that informed the narrative [17]. Data extraction was concurrent with the quality assessment rating process and a second study team member independently verified all extracted data. The study team did not perform a meta‐analysis due to the variability in study samples, in defining characteristics and presentation of interventions, and in definitions of outcome measures.

To draft the PCC framework, two study team members (CMM and JP) met separately to identify domains and sub‐domains for PCC interventions that were identified across all study populations and emerged during the full‐text review. Two other study team members (MD and HZ) independently tested the PCC framework by verifying that the PCC interventions identified in the literature fit within the framework domains and sub‐domains.

### Study quality assessment rating

The methodological quality of the included studies with quantitative components was scored using the Effective Public Health Practice Project (EPHPP) quality assessment tool [18]. Accordingly, randomised and clinical controlled trials were classified as strong quality; those with quality cohort analytic, case–control, cohort design, or interrupted time series were of moderate quality; and studies that used any other method or did not report the method were recorded as weak quality. Using the EPHPP criteria, we adjusted the initial rating related to selection bias, study design, confounders, blinding, data collection, and dropouts. Studies with no weak ratings received an overall ‘strong’ score; studies with one weak rating received an overall “moderate” score; and studies with two or greater weak ratings received an overall ‘weak’ score. A second study team member independently verified all quality assessment ratings.

### Key definitions

Client‐centred care and patient‐centred care are terms widely used in the literature in addition to PCC. The study team opted to use PCC as it considers the whole person, including enablers and barriers that individuals experience in their daily lives which influence their ability to interact with the health facility as a client [19]. We used each study's metrics for adherence, retention, viral load suppression, and any other reported measures to ascertain each intervention's contribution to achieving key HIV health outcomes.

## RESULTS

### Selection of eligible studies

**FIGURE 1:**
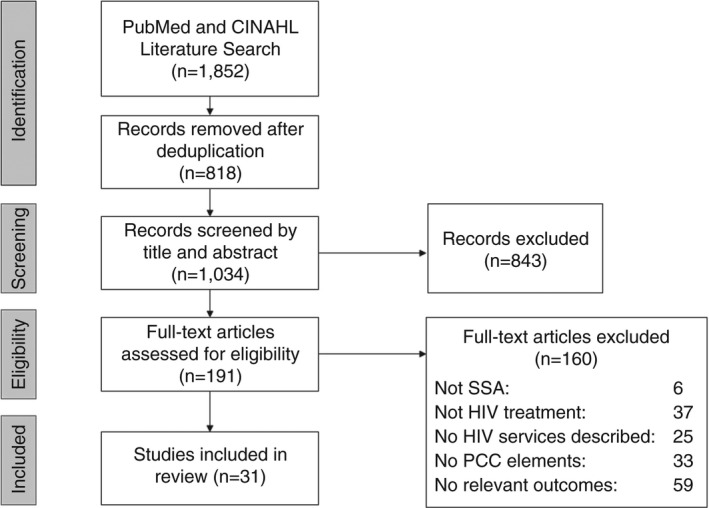
PRISMA flowchart of study selection

Overall, 1852 studies were identified (Figure 1). After the removal of duplicates, the study team examined the title and abstracts of 1034 articles, among which 191 met the criteria for full‐text review. After a full‐text review, 31 studies ultimately met the criteria for inclusion.

### Study characteristics

The studies encompassed 207,673 participants. The largest study included 150,395 individuals and the smallest included 60, with females making up 42%–100% and males 0%–58% of the study populations. PCC interventions targeted women (including pregnant and postpartum) and HIV‐exposed infants (HEI), only male adults, both male and female adults, or adolescents and their parents/caregivers. No studies provided person‐centred interventions for members of key population groups. The studies took place in 12 countries in SSA, with two taking place in multiple countries. Study countries included Kenya (*n* = 7); South Africa (*n* = 7); Uganda (*n* = 5); Zimbabwe (*n* = 5); Mozambique (*n* = 2); and Namibia, Nigeria, Tanzania, Malawi, Rwanda, Zambia, and Lesotho (*n* = 1). Study designs included randomised trial (*n* = 17); retrospective cohort analysis (*n* = 5); program evaluation (*n* = 4), quality improvement assessment (*n* = 2); mixed‐method (*n* = 1); discrete choice experiment (*n* = 1); and cross‐sectional (*n* = 1). Twenty‐eight of the 31 studies implemented combination PCC interventions and 25 studies demonstrated improvements in linkage to care, treatment retention, and/or VS (Table 1).

**TABLE 1:** Summary of PCC interventions from the included studies

Author, year	Country	Description	Quality rating	Population	PCC intervention(s)	Associated outcomes
Boeke et al. 2018	Uganda	Program evaluation of a cohort of lay health workers trained to provide patient follow‐up and counselling.	Moderate	1900 newly diagnosed PLHIV. 60% female/40% male. 75% 19–48 years old, 15% <18 years old.	Lay health workers conduct tracking/documentation of client appointments and follow‐up attempts; make up to two phone calls for missed appointments and home visits to bring clients back to care. Also, provide group education and individual counselling to help clients stay in care.	In smaller facilities (level 3) compared to level 4 facilities, significant linkage increases (*p* < 0.004). % coming to first appointment significantly increased (*p* = 0.03). % coming to ≥4 appointments over 6 months follow‐up significantly increased (*p* = 0.01).
Brown et al. 2019	Uganda and Kenya	Mixed methods to evaluate retention in HIV care among adults (age ≥15) during year 1 of the SEARCH test and treat trial.	Moderate	5683 PLHIV (33%) men, (77%) women. Among men, 86% were ≥30 years; 68% of women were ≥30.	Offer 24‐h telephone access to a clinician; patient‐centered, welcoming, empathetic environment. Flexible clinic hours and locations for ART pick up. Immediate ART initiation; co‐located clinical/ART dispensing; VL monitoring and counselling; co‐located non‐communicable diseases (NCD) care (including hypertension and diabetes); quarterly clinic visits and ART dispensing. Telephone hotline and appointment reminders and tracking to follow‐up missed appointments.	The probability of retention at 1 year was 89.7% for men; 89% for women. Men who were linked to care and initiated ART <30 days were more likely to be retained in care at 1 year.
Cluver et al. 2018	South Africa	Cross‐sectional; interviews and clinical records collected from HIV‐positive adolescents from 53 facilities.	Moderate	1059 adolescents with HIV. 55% female, 45% male. Mean age = 13.8 years. 75% vertically infected with HIV.	Telephone calls to parents and caregivers 3 days prior to the adolescent's appointment to encourage attendance and a phone call to parents and caregivers to report missed appointments.	Factors positively associated with retention: staff with enough time for adolescents; adolescents accompanied to the clinic; enough cash to travel to the clinic; safety during travel; and staff perceived as kind. When more of these factors existed, the greater the likelihood that adolescents were retained in care.
Elul et al. 2017	Mozambique	Randomieed trial, 10 primary health facilities randomly assigned intervention SOC.	Weak	2004 adults ≥18 years. Median age = 34. 64% female, 34% male.	Accelerated ART initiation for clients with POC CD4 cell count <350. Text messages with appointment reminders and health messages to overcome behavioral barriers to seeking care. Cellular airtime cards to offset costs of facility visits in exchange for meeting specific milestones for a maximum amount of $15 USD.	Significant improvements in linkage and retention. 89% in intervention linked to care the same day compared to 16% in the SOC group. 12‐month retention was 58% for intervention and 44% for the SOC group.
Fayorsey et al. 2019	Kenya	Randomised trial with HIV‐positive pregnant women starting ANC at 10 facilities in western Kenya	Strong	340 HIV‐positive pregnant women. Median age 26. Median gestational age 24 weeks. 69% newly diagnosed with HIV during pregnancy.	Hire and train lay health workers to provide home‐ and clinic‐based health education; retention and adherence support; enhanced communication between clients and providers, problem‐solving skills to overcome retention and adherence challenges, and psychosocial support. Provide phone and SMS appointment reminders, follow‐up for missed visits.	Attrition significantly lower at 6 months postpartum in intervention compared to SOC (*p* = 0.04). 222 infants (81.9%) had an HIV PCR test at 6 weeks. Among infants with a negative results, 184 (69.4%) retested at 6 months with no difference between the SOC and intervention.
Fox et al. 2019	South Africa	Randomised evaluation of adherence clubs and observational study of decentralised medication delivery (DMD) in 24 facilities.	Moderate	*n* = 1147. DMD 71% female, 65% ages 30–49. Adherence club (AC) 72% female, 62% ages 30–49.	AC for clinically stable clients. Meet at the health facility or community location for adherence counselling, social support, and pre‐packed medications. Clients meet in groups of 30 every 2–3 months. Pre‐packing and distribution of medications at community pick‐up (CPU) points to decongest clinics and reduce visit burden. Clients are required to attend clinic visits every 6 months.	AC VL: No differences in VL between SOC and intervention. AC retention: Significant improvement in retention in intervention; effects nearly double for men. CPU VL: No differences in sustained VS. CPU retention: Lower retention in intervention.
Fox et al. 2018	South Africa	Randomised evaluation at 12 intervention and 12 control clinics.	Weak	*N* = 863 participants; 61% female.	Enhanced adherence counselling for clients with VL >400. Nurses and counselors provide structured educational counselling to identify barriers and establish goals. Electronic medical records generate reports to identify missed appointments. Trained outreach workers provide telephone follow‐up. Home visits to check on clients and bring them back into care.	Among those with 3‐month VL, suppression was lower in the intervention vs. control arm (15% vs. 35%). At 12 months, enhanced adherence counselling demonstrated no re‐suppression benefit. No evidence of return to care and lower retention in tracing of clients.
Fahey et al. 2020	Tanzania	RCT among newly diagnosed PLHIV assigned SOC; or to receive cash for monthly clinic attendance.	Moderate	*N* = 530 participants over 18 years.	Clients receive cash transfers up to once monthly during the 6 months following enrollment, conditional on attending a clinic visit. Participants could receive a maximum of six transfers (potential $27–60). The cash was to partly cover costs of transportation, food, and lost wages for the time spent at the clinic.	Compared with the SOC, a substantially larger proportion of participants remained in care and achieved VS in both the smaller incentive group (risk difference [RD] 9·8, 95% CI 1·2 to 18·5; *p* = 0.026) and the larger incentive group (RD 13·0, 4·5 to 21·5; *p* = 0.0027). There was no statistically significant difference between incentive arms (RD 3.2, −4.6 to 11.0; *p* = 0.42).
Fatti et al. 2020	Zimbabwe	Randomised trial at 30 health facilities comparing ART delivery methods.	Weak	*N* = 4800. Average age 44–47; 71–74% of study participants female.	Arm 1: Clients received ART every 3 months in CARGs with annual clinical consultations. Arm 2: Clients received ART every 6 months in CARGs with annual clinical consultations.	After 12 months, 1784 (93.0%) SOC, 1265 (94.8%) 3‐month CARG, and 1477 (95.5%) 6 month CARG clients still enrolled. Retention was not significantly higher in the CARG arms (*p* = 0.086) compared to the SOC.
Graves et al. 2018	Uganda	RCT of 46 facilities randomised to family clinic day or the SOC.	Moderate	*n* = 2679 paediatric and adolescent clients 19 months–19 years. Intervention arm: 58% female, 42% male. SOC arm: 57% female, 43% male.	Paediatric and adolescent clients designated specific clinic days; allowed to bring family members for family appointments. Patient flow adapted to prioritise care for families over care for other patients. Expert clients trained to lead health education sessions for adults and caregivers. During each family clinic day, two separate specialised health education sessions were conducted targeting adolescent clients and caregivers of paediatric clients.	Participants from intervention facilities are significantly more likely to adhere to an appointment than SOC (*p*‐ <0.01). Family clinic day attendees were 67% more likely to adhere to their appointment than participants from control facilities (*p* <0.001).
Havlir et al. 2019	Uganda and Kenya	Randomised trial of mobile, 2‐week, multi‐disease health campaigns.	Strong	*n* = 150,395 residents ≥15 years. 55% female, 45% male.	Flexible hours, reduced wait time at clinics.	At 3 years, among all HIV‐positive individuals, VS was 15% higher in the intervention group vs. SOC than in the control group (79% vs. 68%).
Izudi et al. 2018	Uganda	Quality Improvement (QI) design to address low retention rates of HIV‐positive adolescents in a health center.	Weak	*n* = 60 HIV positive adolescents.	Reminder calls to parents/caregivers 3 days prior to adolescent's appointment and to report missed appointments. Adolescent‐only clinics 1 day/month with support groups for adolescents and parents/caregivers to discuss care and treatment challenges.	The number of HIV‐positive adolescents retained in care increased from 34.5% to 96.7% within 3 months and to 96.8% within 6 months. 74.4% of all adolescents who were retained in care accessed VL monitoring and 81.9% attained VS.
Madhombiro et al. 2019	Zimbabwe	RCT testing brief mental illness cognitive behavioral therapy intervention compared to WHO mhGAP intervention for problematic alcohol use in PLHIV.	Moderate	*n* = 80 participants Intervention arm = 42% female, 58% male. Mean age 40 years.	Clients with AUDIT scores that indicate problematic alcohol use randomised to SOC or offered intervention including motivational enhancement therapy, cognitive behavioral therapy, goal setting, and problem‐solving.	There was a statistically significant change in alcohol use in both groups over time (*p* < 0.001) with no difference in the magnitude of change between the groups. No significant CD4 changes or in functioning and quality of life.
Mburu et al. 2019	Kenya	Program evaluation on the effect of Adolescent Package of Care training on VL suppression of 10–19‐year‐olds in 13 facilities.	Weak	*n* = 3540. Pre‐training: 58% female; 61% <15 years old. Post‐training 60% female; 58% <15 years.	Schedule days where only adolescents receive services at the facility. Provide facility‐based training for health providers on adolescent‐friendly services including the use of a 28‐item checklist to assess the needs of adolescents during each visit.	65% virally suppressed during the pre‐training period compared to 72% during the post‐training period (*p* < 0.01). In the pre‐training period, 60% of adolescents received services at clinics offering adolescent clinic days compared to 95% in the post‐training period.
Munyayi et al. 2020	Namibia	Retrospective cohort analysis of adolescents receiving ART.	Moderate	*n* = 385 participants. (78 in the teen club and 307 in SOC).	The teen club meets monthly at a safe space established at the health facility to improve retention in HIV care through psychosocial support, counselling, and health education.	24‐month retention among all adolescents was 90.1%; no significant differences between teen club and SOC (*p* = 0.956).
Myer et al. 2018	South Africa	Randomised trial of postpartum women who initiated ART in ANC, breastfeeding when screened <6 weeks postpartum.	Moderate	*n* = 471 mother‐infant pairs. Average age 28 for both arms.	Postpartum women continue to attend care at MCH clinic while breastfeeding and receive ART and routine infant HIV diagnostic testing. When the mother reports that she has ceased breastfeeding, the nurse‐midwife does a final infant HIV diagnostic test and provides a referral letter for the mother‐baby pair to access nearby ART services.	77% of women in the intervention arm achieved retention in ART services with VL <50 copies/ml at 12 months postpartum, compared to 56% (*n* = 117) of women in SOC arm (*p* < 0.001).
Oyeledun et al. 2017	Nigeria	QI intervention 6 months postpartum of pregnant women who started lifelong ART during pregnancy.	Moderate	*n* = 511 women. Median age 27 at enrolment.	Staff “clock‐in” register to encourage early staff arrival to reduce client‐waiting times. Client satisfaction surveys were administered at the time of client exit from appointment.	There was no difference in retention in care between the intervention and control arms.
Peltzer et al. 2018	South Africa	RCT among pregnant women who received SOC or ‘Protect Your Family’ intervention to examine longitudinal experiences of stigma.	Strong	*n* = 699. SOC = 357. Mean age 28 years.	Lay health workers lead four antenatal and two postnatal group PMTCT sessions. Sessions cover HIV knowledge, vertical transmission prevention, adherence, testing, prevention of transmission and stigma, disclosure, communication with partners, intimate partner violence, infant feeding, safer conception, and family planning including dual methods.	Intervention arm noted reduced stigma from baseline to 12 months.
Pfeiffer et al. 2017	Mozambique	Randomised trial. Pregnant women who tested HIV+ and immediately initiated ART tracked for retention.	Weak	*n* = 761 HIV+ pregnant women. Mean age 25 years. Mean gestational age of 22 weeks at first ANC visit.	Optimise client flow in MCH clinics. Develop SMS texting/phone call protocol and tracking spreadsheet tool with predefined messages for reminders before 30‐day visits, and follow‐up messages to clients 5 days after a missed appointment. Conduct home visits to bring clients back to care.	During control periods, 52.3% of women returned within 5 days before or after their scheduled 30‐day ARV pickup (first refill), compared with 70.8% of women in intervention periods.
Phiri et al. 2017	Malawi	RCT studied facility and community peer support on uptake and retention in Option B+.	Moderate	*n* = 1269 women. Mean age 27.	Mentor/expert mothers (women living with HIV from the community and recently in PMTCT) provided peer support in health facility and community including one‐on‐one support, and weekly clinic‐ or community‐based groups. Mentor mothers contact women within 1 week of a missed appointment via text or telephone (based on client preference), and conduct home visits for clients with missed visits. Home visits include HIV education.	ART uptake was higher in facility‐based and community‐based models in comparison to SOC, but not statistically significant. Among ART initiates, 12‐month retention was similar across the study arms. At 24 months, retention was lower in SOC (66%) compared with facility‐based (80%) or community‐based (83%) models.
Riedel et al. 2018	Rwanda	Retrospective cohort study HIV+ patients in 20 HIV care and treatment sites examined treatment outcomes.	Moderate	*n* = 583 adults ≥15 years; 67% female. Median age 38 years.	Conduct client treatment preparation including educational workshops and involving family and friends of the client in HIV treatment appointments. Offer a range of client‐selected support options, including directly observed and self‐administered therapy.	Overall, 91% of patients suppressed at an average ART duration of 29 months.
Roy et al. 2020	Zambia	Cluster randomised evaluation on effectiveness and implementation of ACs.	Moderate	*n* = 1060. Control 55% female; mean age 41. Intervention 62% female; mean age 42 years.	ACs led by a pharmacy technologist who prepacks and dispenses ART to ~30 clients on ART. Meet every 2 months in the first 6 months and every 3 months thereafter, during evenings or weekends at the facility for medication refills, symptom screening, and group psychosocial support.	Late drug pick up more common in SOC vs. intervention (*p* < 0.0001). Of 597 patients offered AC participation, 99% accepted; 85% attended their first meeting; and 40% attended all meetings.
Ruria et al. 2017	Kenya	Pre/post‐implementation evaluation of the pilot Red Carpet Program implemented in 25 high‐volume boarding schools.	Weak	*n* = 952. Pre‐intervention = 393; 87% female; 52% 15–19 years. Post‐intervention = 559; 86% female; 50% 15–19 years.	In boarding schools: provide counselling on HIV disclosure and sexual and reproductive health; create a supportive environment to ensure ART adherence; create health clubs and provide health education to reduce HIV stigma. Schools offer the storage of HIV medications and link to adolescent‐friendly services in facilities.	100% of participants received peer counselling and psychosocial support. 79% initiated on treatment. Proportion of youth retained on treatment increased from 66% to 90% at 3 months (*p* < 0.001), and from 54% to 99% at 6 months (*p* < 0.001).
Sarna et al. 2019	Kenya	RCT evaluated cell phone counselling intervention to promote retention in care and HIV testing of infants among women accessing PMTCT.	Strong	*n* = 404 pregnant women; average age 24. Intervention arm average age 24. SOC average age 25.	Individualised counselling via cell phone by trained counselors for a maximum of 26 calls during the pregnancy period, and a maximum of 16 calls postpartum. Participants can make additional calls to the counselor during working hours on weekdays to address specific concerns or questions.	Participant retention was significantly higher in the intervention arm than SOC at all three time points (*p* < 0.001). Uptake of infant HIV testing was significantly higher in the intervention arm compared with SOC (*p* < 0.001).
Strauss et al. 2021	Zimbabwe	Discrete choice experiment assessed preferences for ART delivery.	Weak	*n* = 500; 250 females; 50% <30 years. 250 males; 50% <30 years.	Differentiated treatment distribution models include clinic‐based fast track, family and club refill, community‐based outreach, and CARGs.	Preferred services at facility, less frequent visits, individual consultations, shorter waiting times, lower cost delivered by respectful and understanding health staff.
Tapera et al. 2019	Zimbabwe	Retrospective cohort study on HIV care continuum outcomes associated with the peer‐led program for people 0–24.	Moderate	*n* = 15,223 contacts referred for HIV testing by community adolescent treatment supporters.	Trained HIV+ adolescents lead activities for their peers in facility and community settings including co‐facilitating monthly support groups and ART refill groups, and conducting home visits. Send SMS reminders and check‐ins, refer and link people to care, conduct community outreach visits, and co‐facilitate caregiver workshops.	1153 (96.6%) initiated on ART (99% on day of diagnosis). 1151 (99.8%) alive on ART at 6 months; 2 (0.2%) died. 1044 (91%) VL testing at 6 months or later. 1037 (99.3%) were virally suppressed (<1000 copies/m).
Tukei et al. 2020	Lesotho	RCT of community‐ vs. facility differentiated treatment models.	Moderate	*n* = 5336. Facility arm; average age across models 43–48; 61%–72% female.	Community‐based distribution: health worker dispenses ART every 6 months. Community ART groups: clients meet every 3 months one member picks up ART for group and distributes it. Three‐month‐facility pick‐up: SOC with medication dispensation every 3 months at the facility.	Retention not different across arms. After 12 months, 98% virally suppressed in all arms.
van Elsland et al. 2018	South Africa	RCT compared SOC with home‐based adherence intervention in children 0–14.	Strong	*n* = 195. Intervention average age 8, female 59%. Control average age 8, female 57%.	Combined education (information brochure), adherence reinforcement (sticker puzzle), and adherence monitoring (calendar) for caregivers and paediatric clients.	At follow‐up, adherence measured by pill for children using the intervention and controls did not change over time.
Willis et al. 2019	Zimbabwe	RCT evaluated the effect of community services among adolescents living with HIV.	Strong	*n* = 94. Intervention 60% female. Control 62% female.	Trained community adolescent treatment supporters provide weekly home visits with HIV and ART education to other HIV+ adolescents and family/caregivers; monitor adherence; assess wellbeing; make clinic and psychosocial care referrals.	In intervention: linkage to services increased (*p* < 0.001). Retention increased (*p* < 0.001). ART adherence increased (*p* = 0.008). Increase in confidence, self‐esteem, and self‐worth (*p* < 0.001).
Wilson et al. 2019	Kenya	Retrospective cohort evaluated adolescent and young adult engagement in HIV care.	Moderate	*n* = 3662; 54% between 20–24 years; 75% female.	Train clinical staff to provide adolescent‐friendly services and use the Adolescent and Young Adult Care Checklist developed by the Kenyan Government.	Engagement in care significantly higher at facilities where providers trained in adolescent‐friendly care (85.5% vs. 67.7%) and who used the checklist (88.9% vs. 69.2%).
Zanoni et al. 2017	South Africa	Retrospective cohort analysis of retention, VS in HIV+ adolescents/YA	Strong	*n* = 241. Intervention female 57%; median age 17. SOC female 47%; median age 16.	Saturday clinics are designed to reduce school absenteeism. The clinics include pre‐packaged ART dispensing, lunch, and group activities (e.g., dancing, soccer, education, and counselling).	Retention significantly higher in participants attending dedicated adolescent clinic vs. SOC (*p* = 0.018). VS higher among participants attending adolescent clinic vs. SOC (*p* = 0.028).

Abbreviations: AC, adherence club; ANC, antenatal care; ARV, anti‐retrovirals; ART, anti‐retroviral therapy mhGAP, mental health gap action programme; AUDIT, Alcohol Use Disorders Identification Test; CPU, community pick‐up; CARG, community ART‐refill groups; DMD, decentralized medication delivery; MCH, maternal and child health; NCD, non‐communicable disease; PLHIV, persons living with HIV; POC, point of care; PMTCT, prevention of mother to child; QI, Quality Improvement; RCT, randomized controlled trial; SMS, short message service; SOC, standard of care; VL, viral load; WHO, World Health Organization.

### Adolescent PCC interventions

Four studies described support groups for adolescents. In Zimbabwe, community adolescent treatment supporters provided monthly support and ART refill groups for adolescents; co‐facilitated caregiver workshops; provided individual health education and counselling; sent SMS reminders and check‐ins; provided home visits; and referred and linked adolescents to care. Two related studies describe this intervention with outcomes of 97% of adolescents initiating ART, 99% VS at 6 months or later, and improved ART adherence (*p* = 0.008) [13, 14]. A monthly teen club intervention in Namibia provided psychosocial support, HIV counselling, and health education but showed no change in retention [20]. In Uganda, monthly support groups for adolescents and parents/caregivers, telephone appointment reminders, and notification of missed visits contributed to increased retention in care [21].

Two studies focused on retention interventions. A cross‐sectional study in South Africa interviewed adolescents and found a higher likelihood of retention, that increased incrementally with each of the following factors: well‐stocked health facilities, staff perceived as kind and spending enough time with adolescent clients, accompaniment to the health facility, and sufficient funds for clinic visits [22]. A program evaluation in high‐volume boarding schools in Kenya of education and counselling on sexual and reproductive health, adherence, disclosure, and stigma reduction with linkage to adolescent‐friendly services demonstrated an increase in retention at three and 6 months (*p* < 0.001) [23].

Five studies focused on adolescent‐friendly services, special clinics, or home‐based education. Two studies combined adolescent‐friendly training with a related checklist in different settings in Kenya. One showed improved VS, from 65% to 72% (*p* < 0.01) [24], and the other found higher retention at intervention facilities [25]. In South Africa, a Saturday clinic for adolescents with pre‐packaged ART, lunch, and group activities found significantly higher retention than in SOC (*p* < 0.018) [26]. Designated days for paediatric and adolescent clients in Uganda with targeted education for adolescents and their family/caregivers to increase psychosocial, clinical, and life‐skills knowledge increased appointment attendance compared to SOC (*p* < 0.01) [27]. Home‐based education, adherence reinforcement, and monitoring in South Africa showed no change in adherence [28].

### Pregnant and postpartum women and HEI PCC interventions

Five studies employed peer‐based health education and counselling approaches. A study in Malawi, where mentor and expert mothers provided peer‐based support within facilities and homes, showed non‐significant increases in retention at 24 months and increased HEI testing at 12 months but not at 24 months, compared to SOC [29]. In Kenya, a similar intervention found that enrolled women had significantly lower attrition than SOC (*p* < 0.04), but no difference in exclusive breastfeeding or 6‐month HEI testing [30]. In South Africa, a series of clinic‐based sessions on adherence, retention, stigma, interpersonal violence, and other topics for women enrolled in PMTCT contributed to reduced experiences of stigma for pregnant and postpartum women [31]. In Mozambique, an intervention to coordinate care – including home visits, SMS reminders, and individualised counselling – between maternal and child health (MCH) nurses and community health workers and optimise patient flow for immediate ART initiation for newly positive pregnant women, resulted in significant improvements of 30‐day ART pickup and increased return to care [32]. A telephone‐based individualised education and counselling intervention for pregnant and postpartum women contributed to significant increases in retention and HIV infant testing (*p* < 0.001) [33].

One study in Nigeria employed client feedback mechanisms. It examined the impact of client exit interviews on retention outcomes at 6 months postpartum and found no differences in retention between the intervention and SOC arms [34].

One randomised controlled trial (RCT) in South Africa integrated MCH and HIV services, wherein postpartum women remained in MCH services until the cessation of breastfeeding and receipt of the final infant HIV diagnostic test result. Women in the intervention were significantly more likely to be retained in ART services and have suppressed viral loads at 12 months postpartum than SOC (*p* < 0.001) [35].

### General adult population PCC interventions

Three studies included flexibility and efficiency components. In Nigeria, a study showed that introducing a “clock‐in” register for health facility staff to improve on‐time arrival and reduce client wait times did not demonstrate improved retention [34]. Two studies described a mixed‐methods intervention in Uganda and Kenya that offered flexible clinic hours, reduced wait times, and welcoming staff, and demonstrated 89% retention at 12 months for women and 90% for men and increased VS by 15% compared to the SOC [36, 37].

Five studies introduced differentiated treatment delivery models. A three‐armed RCT in Zimbabwe offering a 3‐month visit community ART refill group (CARG) with annual clinic visits, and a 6‐month visit CARG with annual clinic visits found that retention in both groups was slightly but not significantly higher than the SOC (*p* < 0.086) (retention rates: 3 months CARG: 94.8%; 6 months CARG: 95.5%; SOC: 93.0%) [38]. A separate study in Zimbabwe offering a variety of differentiated treatment distribution models including fast‐track, family refill, club refill, community‐based outreach, and CARGs found that clients preferred fewer health facility visits with shorter waiting times and friendly HCW [39]. A study in Zambia trained pharmacy technicians to lead adherence clubs (ACs) every 2–3 months during evenings/weekends and found that late drug pick‐up was significantly more common in the SOC group (*p* < 0.0001) [40]. An RCT in Lesotho comparing community‐based distribution, CARGs, and 3‐month facility pick‐up found that retention did not vary between study arms [41]. In South Africa, a study of pre‐packaged ART distributed during facility‐based AC meetings every 2–3 months showed no change in VS but improved retention, notably doubling among men. The same study showed lower retention and no change in VS in another arm with community pick‐up of pre‐packaged ART [42].

A study in South Africa employed electronic medical records to generate reports on clients who missed appointments and trained outreach workers to provide follow‐up, with no change in return to care or retention [43]. Six studies implemented follow‐up SMS and/or phone calls for clients who missed appointments, all demonstrating improvements in retention at 12 or 24 months [21, 29, 32, 36, 44, 45]. In Uganda and Kenya, HCW placed reminder phone calls in advance of visits and followed up with clients who missed appointments, contributing to 12‐month retention of 88% among men and 87% among women [36]. In Mozambique, a combination intervention approach including SMS appointment reminders and individualised health messages contributed to improved linkage to care (*p* = 0.02) and retention at 12 months (*p* = 0.004) [44].

Two studies introduced conditional incentives. In Tanzania, monthly cash incentives were provided to assist with transport expenses for clients attending HIV treatment services, which resulted in increased retention and VS [46]. In Mozambique, as part of a combination intervention, clients received cellular airtime cards valued at US$5 to offset the costs of appointment attendance, conditional on initiating treatment within 1 month of testing positive, being retained in care at 6 months, and again at 12 months, contributing to improvements in linkage and retention [44].

Six of the studies provided education in health facilities, with two providing education and counselling to enhance adherence and retention. While education did not lead to improvements, group, and individual counselling to improve retention led to significant improvements in adherence to appointment schedules (*p* = 0.01) [43]. In Rwanda, clients prepared for treatment by inviting family and friends to workshops to learn how to support the clients including through directly observed therapy, contributing to a 91% VS rate [47].

In Zimbabwe, an intervention delivering brief mental illness/cognitive behavioural therapy for problematic alcohol use in people with HIV showed reduced alcohol use over time, but no other improvements [48].

### Adult men PCC interventions

A study in Kenya and Uganda provided 24‐h telephone access to a clinician; a person‐centred welcoming and empathetic environment for men within the health facility; co‐located clinical, phlebotomy, and pharmacy; co‐located treatment for hypertension and diabetes; a quarterly visit schedule; and telephone appointment reminders. Findings demonstrated a 90% retention for men at 12 months [36].

### Family PCC interventions

In Uganda, a cluster RCT implemented a family day that adapted patient flow to prioritise families and offered adolescents and parents/caregivers separate health education sessions during the visit. The intervention led to non‐significant improvements in retention in care and significant improvements in adherence to treatment (*p* < 0.001) [27].

Table 2 summarises all person‐centred HIV treatment services offered to each population included in this review.

**TABLE 2:** All PCC interventions for diverse populations found in this systematic review

Population	Continuum outcome	Person‐centered interventions
Adolescents	Retention	Telephone calls to adolescent and family/caregiver with appointment reminders and missed appointment notices; adolescent‐only clinic offered monthly, and support groups for adolescents and parent/caregiver.
	Retention and VL suppression	Community adolescent treatment supporters facilitate support and ART refill groups and provide counselling, home visits, and missed appointment follow up; Saturday clinics with pre‐packaged ART; group activities including counselling and sports; health workers use ministry of health checklist to provide adolescent‐friendly services during adolescent clinic.
	Linkage and retention	Boarding school students offered HIV and SRH counselling, ART storage, health clubs, and treatment linkage.
Pregnant/postpartum women, HEI	Retention and infant testing	Lay health workers provide clinic‐ and home‐based education, texts with appointment reminders and missed visit follow‐up; postpartum women receive HIV care at MCH clinic until breastfeeding cessation and final infant HIV test; individual counselling to pregnant and postpartum women provided via telephone.
General adult population	Retention	Expert patients provide group and individual education/counselling, phone calls and home visits; community ART refill groups, fast‐track, club refills, family refills.
	Linkage and retention	Expedited ART initiation for people with CD4<350; text appointment reminders with integrated health messages; and cellular airtime to offset clinic visit costs.
	Retention and VL suppression	Adherence clubs in facilities/communities with ART dispensation; community pick‐up with pre‐packaged ART; enhanced adherence counselling for clients with VL >400; EMR reports to follow‐up missed visits.
	VL suppression	Cash transfers to cover clinic transport costs; flexible hours, reduced waiting time, and friendly staff; treatment preparation for clients and family members with education workshops to enhance retention in treatment and to provide directly observed therapy.
Adult men	Retention and VL suppression	Flexible clinic hours; 24‐h question hotline; differentiated ART distribution in community settings; reduced clinic visits; telephone appointment reminders.
Families	Retention	Family clinic days scheduled; individual counselling for parents and adolescents.

Abbreviations: ART, anti‐retroviral therapy; EMR, electronic medical record; MCH, maternal and child health; PLHIV, persons living with HIV; SRH, sexual and reproductive health; VL, viral load.

### PCC framework

**FIGURE 2:**
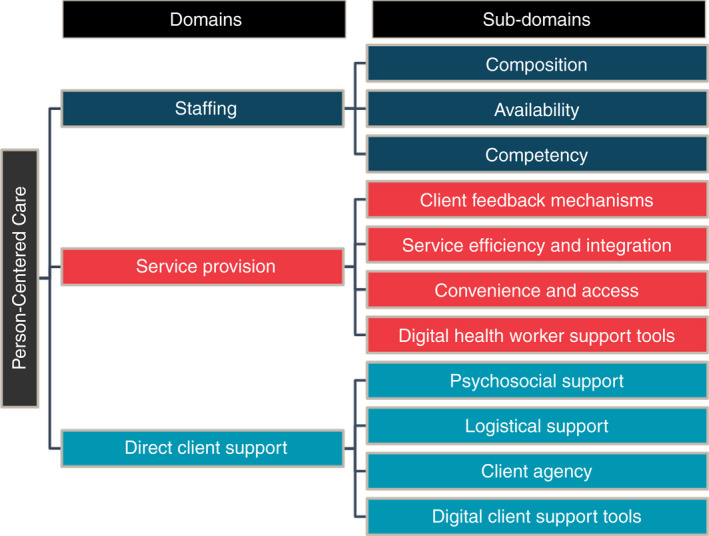
PCC Framework. The figure depicts the three domains and 11 sub‐domains of PCC for HIV treatment identified through this review

Further synthesis of the interventions led to the identification of three broad domains of PCC implemented across all of the study populations: staffing, service provision standards, and direct client support services. Taken collectively, these domains and their 11 associated sub‐domains constitute a framework for defining PCC (see Figure 2).

### Quality assessment of quantitative studies

Appraisal of the studies with quantitative methods components demonstrated that seven received a strong global quality rating; 16 a moderate global quality rating; and eight a weak global quality rating. Weak components included selection bias [48]; study design [20, 21, 40]; confounders [21, 23, 24, 33, 40, 47]; blinding [31, 39, 43, 45]; data collection [12, 20, 21, 23, 24, 25, 27, 29, 33, 35, 36, 39, 41, 42, 44, 45, 46, 48, 49]; and withdrawals and drop‐outs [33, 43]. The [Link], [Link] section contains detailed quality rating scores for each study.

## DISCUSSION

This systematic review describes a wide variety of population‐specific interventions that align with WHO, PEPFAR, and other definitions for PCC. These interventions increase convenience, make services supportive and accessible, provide welcoming services to diverse populations, and engage communities and stakeholders [6]. The resulting framework refocuses and defines PCC for health facilities providing HIV treatment irrespective of the target population, as they strive to staff services appropriately, set optimal service provision standards, and provide support services directly to clients. By providing a consistent classification of PCC interventions, this preliminary framework will facilitate assessment and reinforce comprehensive PCC.

Several domains and sub‐domains within the proposed framework are interrelated and many of the included studies described combination interventions. Not all interventions demonstrated improvements along the care continuum; interventions in certain sub‐domains may be necessary but insufficient to improve outcomes. Some interventions may be more successful within certain contexts, with different sub‐populations, or in combination with different interventions. In the context of global declines in HIV funding, some interventions may lead to more efficient use of resources and thus seek to simply demonstrate non‐inferior health outcomes.

### Staffing

Previous research on PCC for the control of chronic disease highlights the importance of HCW collaborating with clients to gather medical, personal, and social histories; make joint decisions; and document clients' preferences to allow for transparency and to facilitate continuity of care [50].

The composition sub‐domain indicates that clinic staff are culturally competent and speak the primary language(s) of the communities that they serve, including representation of specific sub‐populations. Four included studies recruited lay cadres of PLHIV (e.g., mentor mothers, expert clients, or peer navigators) from the community to assist with client tracing and health education, demonstrating improvements across the care continuum [30, 31, 40, 45]. This approach is widely proven to enhance HIV service access and the quality of care [49, 51, 52].

The availability sub‐domain stipulates that allocated health facility positions are filled, and that staff are available to provide services throughout health facility operating hours to facilitate reduced client wait times, and responsiveness to client questions. Two included studies focused on staff availability [34, 36]. Though only one demonstrated improvement, other studies identify staff availability as foundational to effective health care [53].

The competency sub‐domain pertains to the specific staff qualifications to meet job description requirements, including cultural and linguistic competency and customer services approaches. Four included studies focused on strengthening staff competency, with all contributing to improvement [22, 23, 24, 25].

### Service provision standards

Attributes of patient‐centred primary care that were found to apply to HIV treatment include respect for patient preferences and integrated and coordinated care [54].

The client feedback mechanism sub‐domain includes the participation of clients in planning, day‐to‐day management, and evaluation of HIV treatment services using a variety of mechanisms for client feedback intentionally gathered and routinely reviewed. One included study examined the impact of client exit interviews on retention [34]. Though it did not identify improvement, client feedback mechanisms are integral components within other PCC delivery frameworks and hold potential for people to shape services based upon their own needs and contribute ideas for service improvement [54].

The service efficiency and integration sub‐domain include service models that reduce the duration and number of required visits for individuals and families: providing multiple health services during a single visit; enhancing patient flow while safeguarding confidentiality through visual and aural privacy; and coordinating family visits. Six studies included service efficiency and integration components all leading to significant improvements in retention and VS [27, 32, 35, 36, 44, 45].

The convenience and access sub‐domain pertain to adapting health service locations to reach clients where they are (e.g., differentiated treatment models) and flexible timing of visits (e.g., weekend, evening, and special clinics for different populations). Twelve included studies implemented convenience and access interventions, with many showing small or no improvement, while weekend clinics, flexible clinic hours, and reduced wait times led to increased adherence, retention, and VS [21, 24, 26, 27, 36, 37, 38, 39, 40, 41, 42, 44].

The digital tools to support the HCW sub‐domain include HCW training via information technology mechanisms to support PCC; digital job aids; clinical decision support; access to client records to generate reports of upcoming and missed appointments; and tele‐medicine and ‐counselling. Four included studies used digital tools to support HCW, with three demonstrating improvements in retention [21, 30, 36, 43].

### Direct client support services

This domain includes client information and education, emotional support, and family and friend involvement [55, 56]. These aspects translate to the PCC framework for HIV treatment, and all contribute to building client agency.

The psychosocial support sub‐domain includes peer support groups, caregiver and treatment buddies, and disclosure support. Five included studies provided psychosocial support interventions; most were included within a combination intervention that demonstrated improvements while one not part of a combination did not identify any change in retention [14, 20, 23, 30, 40]. Two studies included family and peer support, with both achieving good outcomes [13, 47].

The logistical support sub‐domain includes transport, travel vouchers, food support, childcare, cash, or other (vouchers and cell phone credit) incentives to support client engagement with treatment services. Three studies included logistical interventions, all contributing to improvements in linkage, retention, and VS [22, 44, 46].

The client‐agency sub‐domain includes providing client education including making medical information understandable and actionable and ensuring that clients understand their rights. Eleven studies included client‐agency components, with most contributing to significant improvements [14, 23, 27, 28, 29, 30, 31, 32, 33, 43, 48].

The digital tools to support clients' sub‐domain include appointment/adherence reminders, appointment scheduling, and question hotlines, and client access to health and lab records. Six studies used digital technology with all contributing to improvements [21, 29, 30, 32, 36, 44].

### Limitations and future research

While this review presents a framework for PCC for facility‐level HIV treatment, there are some important limitations. This review presents a preliminary framework. It must be supplemented by perspectives of PLHIV and HCW, to be solicited during a forthcoming validation process, and will be published separately. The domains and sub‐domains presented are not necessarily mutually exclusive, but important for programmers to consider. The study team chose to include only those studies that reported outcomes to understand the potential importance of a given person‐centred intervention. There was a dearth of studies that highlighted PCC for members of key populations, and that included mental health as a critical component of PCC, indicating critical gaps in the literature that require further study. Future studies on PCC may also consider the inclusion of detailed cost analysis for PCC interventions. This framework does not consider HIV prevention or community treatment settings, limiting the scope of application. Finally, the methodologic rigor of the studies selected for the review is also a limitation, as very few (6 out of 31) had a “Strong” quality rating.

### Conclusions

The study team conducted a very broad search given the varying terminology and lack of consensus in this relatively new area of study. This study can contribute to a more consistent classification and assessment of interventions. The framework will inform the development of a PCC assessment tool that will articulate core performance measures. Operationalisation and validation of the framework and tool in diverse settings and among diverse populations of PLHIV and HCW will further contribute to the knowledge base of PCC within HIV treatment settings in SSA.

## Supporting information

File S1

File S2
