# Supplementary material for: Systematic review: Development of a person‐centered care framework within the context of HIV treatment settings in sub‐Saharan Africa
Source: Trop Med Int Health. 2022 Apr 1;27(5):479–93. doi: 10.1111/tmi.13746 (PMC9324124; doi:10.1111/tmi.13746)
Supplement: Supplementary file 1 — File S1 [file TMI-27-479-s002.pdf]

Search Strategy

PubMed

Search date: April 23, 2021
All fields, MeSH Terms
Filters: English only, published beginning in January 2016 until April 2021.

CINAHL
Search date: April 23, 2021
Abstract
Filters: English only, published beginning in January 2016 until April 2021.

Searches:
((Person centered care) OR (Client centered care)) AND (HIV)

(Differentiated service delivery) AND (HIV)

(Adolescent friendly) AND (HIV)

(Male friendly) AND (HIV)

((Pregnant women) AND (Friendly)) AND (HIV)

(Key population friendly) AND (HIV)

(((((((Men) AND (HIV treatment)) OR (Access)) OR (engagement)) OR (retention)) OR (uptake)) OR (adherence)) OR (access)

(((((((Pregnant women) AND (HIV treatment)) OR (PMTCT)) AND (Access)) OR (Engagement)) OR (Uptake)) OR (adherence)) OR (retention)

(((((((Women of childbearing age) AND (HIV treatment)) OR (PMTCT)) AND (Access)) OR (Engagement)) OR (Uptake)) OR (adherence)) OR (retention)

((((((((Adolescent) OR (teenager)) OR (youth)) AND (HIV treatment)) AND (uptake)) OR (retention)) OR (access)) OR (adherence)) OR (engagement)

((((((((Child) OR (pediatric) AND (HIV treatment)) AND (uptake)) OR (retention)) OR (access)) OR (adherence)) OR (engagement)
