# Supplementary material for: Systematic review: Development of a person‐centered care framework within the context of HIV treatment settings in sub‐Saharan Africa
Source: Trop Med Int Health. 2022 Apr 1;27(5):479–93. doi: 10.1111/tmi.13746 (PMC9324124; doi:10.1111/tmi.13746)
Supplement: Supplementary file 2 — File S2 [file TMI-27-479-s001.pdf]

		Boeke et al., 2018		Brown et al., 2019		Cluver et al., 2018	
	Team member	MD	HZ	MD	HZ	MD	HZ
Selection Bias	Represent population	Somewhat likely	Somewhat likely	Very likely	Very likely	Somewhat likely	Somewhat likely
	% agreed to participate	Can't tell	Can't tell	80-100%	80-100%	80-100%	80-100%
	Section Rating	Moderate	Moderate	Strong	Strong	Moderate	Moderate
Study Design	Study design	Cohort (pre/post)	Cohort (pre/post)	Mixed methods within a RCT	Mixed methods within RCT	Cross sectional	Cross sectional,
	Randomized	Yes	Yes	Yes	Yes	No	No
	Randomization described	No	No	Yes	Yes	No	No
	Randomization appropriate	No	No	Yes	Yes	No	No
	Section Rating	Moderate	Moderate	Strong	Strong	Weak	Weak
Confoun-ders	Difference between groups pre-intervention	Yes	Yes	Yes	Yes	Yes	No
	% of confounders controlled for	80-100%	80-100%	Yes	80-100%	Yes	80-100%
	Section Rating	Strong	Strong	Strong	Strong	Strong	Strong
Blinding	Assessors aware of intervention/exposure	Can't tell	Can’t tell	Can't tell	Can't tell	N/A	N/A
	Participants aware of research question	Can't tell	Can't tell	Can't tell	Can't tell	N/A	N/A
	Section Rating	Moderate	Moderate	Moderate	Moderate	N/A	N/A
Data Collection	Data collection methods valid	Can't tell	Can't tell	Can't tell	Can't tell	Yes	Yes
	Data collection tools shown to be reliable	Can't tell	Can't tell	Can't tell	Can't tell	Yes	Yes
	Section Rating	Weak	Weak	Weak	Weak	Strong	Strong
Withdrawals and Drop-outs	Withdrawals/drop-outs reported in #s/reasons	Yes	Yes	Yes	Yes	Yes	Yes
	% completing the study	80-100%	80-100%	80-100%	80-100%	80-100%	80-100%
	Section Rating	Strong	Strong	Strong	Strong	Strong	Strong
Final Score	Reviewer discrepancy	N		No		Y - Blinding	
	Reason for discrepancy					Mismatch with blinding	
	**Final global rating**	**Moderate**		**Moderate**		**Moderate**	

		Elul et al., 2017		Fayorsey et al., 2019		Fox et al., 2019	
	Team member	MD	HZ	MD	HZ	MD	HZ
Selection Bias	Represent population	Very likely	Yes	Very likely	Very likely	Very likely	Very likely
	% agreed to participate	80-100%	80-100%	80-100%	80-100%	80-100%	80-100%
	Section Rating	Strong	Strong	Strong	Strong	Strong	Strong
Study Design	Study design	RCT	RCT	Randomized trial	Randomized trial	Randomized trial	Randomized trial and Observational Study
	Randomized	Yes	Yes]	Yes	Yes	Yes	Yes
	Randomization described	Yes	Yes	Yes	Yes	Yes	Yes
	Randomization appropriate	Yes	Yes	Yes	Yes	Yes	Yes
	Section Rating	Strong	Strong	Strong	Strong	Strong	Strong
Confoun-ders	Difference between groups pre-intervention	Yes	Yes	Yes	Yes	Yes	Yes
	% of confounders controlled for	80-100%	80-100%	80-100%	80-100%	80-100%	80-100%
	Section Rating	Strong	Strong	Strong	Strong	Strong	Strong
Blinding	Assessors aware of intervention/exposure	Yes	Yes	Yes	Yes	Yes	Yes
	Participants aware of research question	Yes	Yes	Yes	Yes	Yes	Yes
	Section Rating	Weak	Weak	Weak	Weak	Weak	Weak
Data Collection	Data collection methods valid	Can't tell	Can't tell	Yes	Yes	Yes	Yes
	Data collection tools shown to be reliable	Can't tell	Can't tell	Can't tell	Can't tell	Can't tell	Can't tell
	Section Rating	Weak	Weak	Moderate	Moderate	Moderate	Moderate
Withdrawals and Drop-outs	Withdrawals/drop-outs reported in #s/reasons	Yes	Yes	Yes	Yes	Yes	Yes
	% completing the study	80-100%	80-100&	80-100%	80-100%	80-100%	80-100%
	Section Rating	Strong	Strong	Strong	Strong	Strong	Strong
Final Score	Reviewer discrepancy	No		No		No	
	Reason for discrepancy						
	**Final global rating**	**Weak**		**Strong**		**Moderate**	

		Fox et al., 2018		Fahey et al., 2017		Fatti et al., 2020	
	Team member	MD	HZ	MD	HZ	MD	HZ
Selection Bias	Represent population	Very likely	Very likely	Very likely	Very likely	Very likely	Very likely
	% agreed to participate	80-100%	80-100%	80-100%	80-100%	80-100%	80-100%
	Section Rating	Strong	Strong	Strong	Strong	Strong	Strong
Study Design	Study design	Cluster randomized evaluation	Cluster randomized evaluation	Randomized trial	Randomized trial	Cluster randomized trial	Cluster randomized trial
	Randomized	Yes	Yes	Yes	Yes	Yes	Yes
	Randomization described	Yes	Yes	Yes	Yes	Yes	Yes
	Randomization appropriate	Yes	Yes	Yes	Yes	Yes	Yes
	Section Rating	Strong	Strong	Strong	Strong	Strong	Strong
Confoun-ders	Difference between groups pre-intervention	Yes	Yes	Yes	Yes	Yes	Yes
	% of confounders controlled for	80-100%	80-100%	<60%	<60%	80-100%	80-100%
	Section Rating	Strong	Strong	Weak	Weak	Strong	Strong
Blinding	Assessors aware of intervention/exposure	Can't tell	Can't tell	Can't tell	Can't tell	No	Yes
	Participants aware of research question	Can't tell	Can't tell	Can't tell	Can't tell	Yes	Yes
	Section Rating	Moderate	Moderate	Moderate	Moderate	Weak	Weak
Data Collection	Data collection methods valid	Can't tell	Can't tell	Yes	Yes	Can't tell	Can't tell
	Data collection tools shown to be reliable	Can't tell	Can't tell	Yes	Yes	Can't tell	Can't tell
	Section Rating	Weak	Weak	Strong	Strong	Weak	Weak
Withdrawals and Drop-outs	Withdrawals/drop-outs reported in #s/reasons	Yes	Yes	Yes	Yes	Yes	Yes
	% completing the study	60-79%	Total<60%	80-100%	80-100%	80-100%	80-100%
	Section Rating	Moderate	Weak	Strong	Strong	Strong	Strong
Final Score	Reviewer discrepancy	Yes		No		Yes	
	Reason for discrepancy	Miscalculation					
	**Final global rating**	**Weak**		**Moderate**		**Weak**	

		Graves et al., 2018		Havlir et al., 2019		Izudi et al., 2018	
	Team member	MD	HZ	MD	HZ	MD	HZ
Selection Bias	Represent population	Very likely	Very likely	Very likely	Very likely	Somewhat likely	Somewhat likely
	% agreed to participate	80-100%	80-100%	80-100%	80-100%	80-100%	80-100%
	Section Rating	Strong	Strong	Strong	Strong	Moderate	Moderate
Study Design	Study design	RCT	RCT	RCT	RCT	QI design	QI design
	Randomized	Yes	Yes	Yes	Yes	No	No
	Randomization described	Yes	Yes	Yes	Yes	No	No
	Randomization appropriate	Yes	Yes	Yes	Yes	No	No
	Section Rating	Strong	Strong	Strong	Strong	Weak	Weak
Confoun-ders	Difference between groups pre-intervention	Yes	Yes	Yes	Yes	No	No
	% of confounders controlled for	80-100%	80-100%	80-100%	80-100%	No	No
	Section Rating	Strong	Strong	Strong	Strong	Weak	Weak
Blinding	Assessors aware of intervention/exposure	Can't tell	Can't tell	Can't tell	Can't tell	Can't tell	Yes - by virtue of the design
	Participants aware of research question	Can't tell	Can't tell	Can't tell	Can't tell	Can't tell	Can't tell
	Section Rating	Moderate	Moderate	Moderate	Moderate	Moderate	Moderate
Data Collection	Data collection methods valid	Can't tell	Can't tell	Yes	Yes	Can't tell	Can't tell
	Data collection tools shown to be reliable	Can't tell	Can't tell	Yes	Yes	Can't tell	Can't tell
	Section Rating	Weak	Weak	Strong	Strong	Weak	Weak
Withdrawals and Drop-outs	Withdrawals/drop-outs reported in #s/reasons	N/A	N/A	Yes	Yes	Yes	Yes
	% completing the study	N/A	N/A	80-100%	80-100%	80-100%	80-100%
	Section Rating	N/A	N/A	Strong	Strong	Strong	Strong
Final Score	Reviewer discrepancy	No		No		No	
	Reason for discrepancy						
	**Final global rating**	**Moderate**		**Strong**		**Weak**	

		Madhombiro et al., 2019		Mburu et al., 2019		Munyayi et al., 2020	
	Team member	MD	HZ	Very likely	Very likely	MD	HZ
Selection Bias	Represent population	Not likely	Not likely	80-100%	80-100%	Somewhat likely	Somewhat likely
	% agreed to participate	80-100%	80-100%	Strong	Strong	N/A	N/A
	Section Rating	Weak	Weak	Cohort (pre/post)	Cohort (pre/post)	Moderate	Moderate
Study Design	Study design	Cohort analytic	Cohort analytic	Yes	No	Retrospective cohort analysis	Retrospective cohort analysis
	Randomized	Yes	Yes	Yes	No	No	No
	Randomization described	Yes	Yes	Yes	No	No	No
	Randomization appropriate	Yes	Yes	Moderate	Moderate	No	No
	Section Rating	Moderate	Moderate	Yes	Yes	Moderate	Moderate
Confoun-ders	Difference between groups pre-intervention	Yes	Yes	<60%	<60%	Yes	Yes
	% of confounders controlled for	N/A	N/A	Weak	Weak	80-100%	80-100%
	Section Rating	Strong	Strong	Can't tell	Can't tell	Strong	Strong
Blinding	Assessors aware of intervention/exposure	Can't tell	Can't tell	Can't tell	Can't tell	Can't tell	Yes
	Participants aware of research question	Can't tell	Can't tell	Moderate	Moderate	Can't tell	Can't tell
	Section Rating	Moderate	Moderate	Can't tell	Can't tell	Moderate	Moderate
Data Collection	Data collection methods valid	Yes	Yes	Can't tell	Can't tell	Can't tell	Can't tell
	Data collection tools shown to be reliable	Yes	Yes	Weak	Weak	Can't tell	Can't tell
	Section Rating	Strong	Strong	No	No	Weak	Weak
Withdrawals and Drop-outs	Withdrawals/drop-outs reported in #s/reasons	Yes	Yes	N/A	N/A	N/A	N/A
	% completing the study	60-79%	60-79%	Weak	Weak	N/A	N/A
	Section Rating	Moderate	Moderate	Very likely	Very likely	N/A	N/A
Final Score	Reviewer discrepancy	Yes		No		No	
	Reason for discrepancy	Miscalculation					
	**Final global rating**	**Moderate**		**Weak**		**Moderate**	

		Myer et al., 2018		Oyeledun et al., 2017		Peltzer et al., 2018	
	Team member	MD	HZ	MD	HZ	MD	HZ
Selection Bias	Represent population	Very likely	Very likely	Very likely	Very likely	Very likely	Very likely
	% agreed to participate	80-100%	80-100%	80-100%	80-100%	80-100%	80-100%
	Section Rating	Strong	Strong	Strong	Strong	Strong	Strong
Study Design	Study design	Randomized trial	Randomized trial	Randomized trial	Randomized trial	Randomized trial	Randomized trial
	Randomized	Yes	Yes	Yes	Yes	Yes	Yes
	Randomization described	Yes	Yes	Yes	Yes	Yes	Yes
	Randomization appropriate	Yes	Yes	Yes	Yes	Yes	Yes
	Section Rating	Strong	Strong	Strong	Strong	Strong	Strong
Confoun-ders	Difference between groups pre-intervention	Yes	Yes	Yes	Yes	Yes	Yes
	% of confounders controlled for	80-100%	80-100%	80-100%	80-100%	80-100%	80-100%
	Section Rating	Strong	Strong	Strong	Strong	Strong	Strong
Blinding	Assessors aware of intervention/exposure	Yes	Yes	Can't tell	Can't tell	Can't tell	Can't tell
	Participants aware of research question	Can't tell	Can't tell	Can't tell	Can't tell	Can't tell	Can't tell
	Section Rating	Moderate	Moderate	Moderate	Moderate	Moderate	Moderate
Data Collection	Data collection methods valid	Can't tell	Can't tell	Can't tell	Can't tell	Yes	Yes
	Data collection tools shown to be reliable	Can't tell	Can't tell	Can't tell	Can't tell	Yes	Yes
	Section Rating	Weak	Weak	Weak	Weak	Strong	Strong
Withdrawals and Drop-outs	Withdrawals/drop-outs reported in #s/reasons	Yes	Yes	Yes	Yes	Yes	Yes
	% completing the study	80-100%	80-100%	60-79%	60-79%	60-79%	60-79%
	Section Rating	Strong	Strong	Moderate	Moderate	Moderate	Moderate
Final Score	Reviewer discrepancy	No		No		No	
	Reason for discrepancy						
	**Final global rating**	**Moderate**		**Moderate**		**Strong**	

		Pfeiffer et al., 2017		Phiri et al., 2017		Riedel et al., 2018	
	Team member	MD	HZ	MD	HZ	MD	HZ
Selection Bias	Represent population	Very likely	Very likely	Very likely	Very likely	Very likely	Very likely
	% agreed to participate	80-100%	80-100%	80-100%	80-100%	80-100%	80-100%
	Section Rating	Strong	Strong	Strong	Strong	Strong	Strong
Study Design	Study design	Stepped-wedge RCT	Randomized trial	3-arm cluster randomized controlled trial	Randomized controlled trial	Retrospective cohort study	Retrospective cohort study
	Randomized	Yes	Yes	Yes	Yes	Yes	Yes
	Randomization described	Yes	Yes	Yes	Yes	Yes	Yes
	Randomization appropriate	Yes	Yes	Yes	Yes	Yes	Yes
	Section Rating	Strong	Strong	Strong	Strong	Moderate	Moderate
Confoun-ders	Difference between groups pre-intervention	Yes	Yes	Yes	Yes	Yes	Yes
	% of confounders controlled for	<60%	<60%	80-100%	80-100%	80-100%	80-100%
	Section Rating	Weak	Weak	Strong	Strong	Strong	Strong
Blinding	Assessors aware of intervention/exposure	Can't tell	Can't tell	Can't tell	Can't tell	Can't tell	Can't tell
	Participants aware of research question	Can't tell	Can't tell	Can't tell	Can't tell	Can't tell	Can't tell
	Section Rating	Moderate	Moderate	Moderate	Moderate	Moderate	Moderate
Data Collection	Data collection methods valid	Can't tell	Can't tell	Can't tell	Can't tell	Can't tell	Can't tell
	Data collection tools shown to be reliable	Can't tell	Can't tell	Can't tell	Can't tell	Can't tell	Can't tell
	Section Rating	Weak	Weak	Weak	Weak	Weak	Weak
Withdrawals and Drop-outs	Withdrawals/drop-outs reported in #s/reasons	No	No	Yes	Yes	Yes	Yes
	% completing the study	N/A	N/A	80-100%	80-100%	80-100%	80-100%
	Section Rating	Weak	Weak	Strong	Strong	Strong	Strong
Final Score	Reviewer discrepancy	No		No		No	
	Reason for discrepancy						
	**Final global rating**	**Weak**		**Moderate**		**Moderate**	

		Roy et al., 2020		Ruria et al., 2017		Sarna et al., 2019	
	Team member	MD	HZ	MD	HZ	MD	MD
Selection Bias	Represent population	Very likely	Very likely	Somewhat likely	Somewhat likely	Very likely	Very likely
	% agreed to participate	80-100%	80-100%	80-100%	80-100%	80-100%	80-100%
	Section Rating	Strong	Strong	Moderate	Moderate	Strong	Strong
Study Design	Study design	Randomized trial	Randomized trial	Cohort	Cohort	Randomized controlled study	Randomized controlled study
	Randomized	Yes	Yes	No	No	Yes	Yes
	Randomization described	Yes	Yes	No	No	Yes	Yes
	Randomization appropriate	Yes	Yes	No	No	Yes	Yes
	Section Rating	Strong	Strong	Moderate	Moderate	Strong	Strong
Confoun-ders	Difference between groups pre-intervention	Yes	Yes	Yes	Yes	Yes	Yes
	% of confounders controlled for	<60%	<60%	<60%	<60%	80-100%	80-100%
	Section Rating	Weak	Weak	Weak	Weak	Strong	Strong
Blinding	Assessors aware of intervention/exposure	Can't tell	Can't tell	Can't tell	Can't tell	No	No
	Participants aware of research question	Can't tell	Can't tell	Can't tell	Can't tell	No	No
	Section Rating	Moderate	Moderate	Moderate	Moderate	Strong	Strong
Data Collection	Data collection methods valid	Yes	Yes	Can't tell	Can't tell	Yes	Yes
	Data collection tools shown to be reliable	Yes	Yes	Can't tell	Can't tell	Yes	Yes
	Section Rating	Strong	Strong	Weak	Weak	Strong	Strong
Withdrawals and Drop-outs	Withdrawals/drop-outs reported in #s/reasons	Yes	Yes	Yes	Yes	Yes	Yes
	% completing the study	80-100%	80-100%	80-100%	80-100%	80-100%	80-100%
	Section Rating	Strong	Strong	Strong	Strong	Strong	Strong
Final Score	Reviewer discrepancy	No		No		No	
	Reason for discrepancy						
	**Final global rating**	**Moderate**		**Weak**		**Strong**	

		Strauss et al., 2021		Tapera et al., 2019		Tukei et al., 2020	
	Team member	MD	HZ	MD	HZ	MD	HZ
Selection Bias	Represent population	Somewhat likely	Somewhat likely	Somewhat likely	Somewhat likely	Very likely	Very likely
	% agreed to participate	Can't tell	Can't tell	80-100%	80-100%	80-100%	80-100%
	Section Rating	Moderate	Moderate	Moderate	Moderate	Strong	Strong
Study Design	Study design	Discrete choice experiment	Discrete choice experiment	Retrospective cohort study	Retrospective cohort study	Cluster randomized trial	Randomized trial
	Randomized	No	No	No	No	Yes	Yes
	Randomization described	No	No	No	No	Yes	Yes
	Randomization appropriate	No	No	No	No	Yes	Yes
	Section Rating	Weak	Weak	Moderate	Moderate	Strong	Strong
Confoun-ders	Difference between groups pre-intervention	Yes	Yes	Yes	Yes	Yes	Yes
	% of confounders controlled for	>80%	80-100%	60-79%	60-79%	60-79%	60-79%
	Section Rating	Strong	Strong	Moderate	Moderate	Moderate	Moderate
Blinding	Assessors aware of intervention/exposure	N/A	N/A	Can't tell	Can't tell	Can't tell	Can't tell
	Participants aware of research question	N/A	N/A	Can't tell	Can't tell	Can't tell	Can't tell
	Section Rating	N/A	N/A	Moderate	Moderate	Moderate	Moderate
Data Collection	Data collection methods valid	No	No	Can't tell	Can't tell	Can't tell	Can't tell
	Data collection tools shown to be reliable	No	No	Can't tell	Can't tell	Can't tell	Can't tell
	Section Rating	Weak	Weak	Weak	Weak	Weak	Weak
Withdrawals and Drop-outs	Withdrawals/drop-outs reported in #s/reasons	N/A	N/A	N/A	N/A	Yes	Yes
	% completing the study	N/A	N/A	N/A	N/A	>80%	60-79%
	Section Rating	N/A	N/A	N/A	N/A	Strong	Strong
Final Score	Reviewer discrepancy	No		No		No	
	Reason for discrepancy						
	**Final global rating**	**Weak**		**Moderate**		**Moderate**	

		van Esland et al., 2017		Wills et al., 2019		Wilson et al., 2019	
	Team member	MD	HZ	MD	HZ	MD	HZ
Selection Bias	Represent population	Very likely	Very likely	Very likely	Very likely	Very likely	Very likely
	% agreed to participate	80-100%	80-100%	80-100%	80-100%	N/A	N/A
	Section Rating	Strong	Strong	Strong	Strong	Strong	Strong
Study Design	Study design	Randomized controlled study	Randomized controlled study	Randomized trial	Randomized trial	Retrospective cohort study	Retrospective cohort study
	Randomized	Yes	Yes	Yes	Yes	No	No
	Randomization described	Yes	Yes	Yes	Yes	No	No
	Randomization appropriate	Yes	Yes	Yes	Yes	No	No
	Section Rating	Strong	Strong	Strong	Strong	Moderate	Moderate
Confoun-ders	Difference between groups pre-intervention	Yes	Yes	Yes	Yes	Yes	Yes
	% of confounders controlled for	>80%	60-79%	60-79%	60-79%	60-80%	60-79%
	Section Rating	Strong	Strong	Moderate	Moderate	Moderate	Moderate
Blinding	Assessors aware of intervention/exposure	Can't tell	Can't tell	Can't tell	Can't tell	N/A	N/A
	Participants aware of research question	Can't tell	Can't tell	Can't tell	Can't tell	N/A	N/A
	Section Rating	Moderate	Moderate	Moderate	Moderate	N/A	N/A
Data Collection	Data collection methods valid	Yes	Yes	Yes	Yes	Can't tell	Can't tell
	Data collection tools shown to be reliable	Yes	Yes	Yes	Yes	Can't tell	Can't tell
	Section Rating	Strong	Strong	Strong	Strong	Weak	Weak
Withdrawals and Drop-outs	Withdrawals/drop-outs reported in #s/reasons	Yes	Yes	Yes	Yes	N/A	N/A
	% completing the study	60-79%	60-79%	>80%	60-79%	N/A	N/A
	Section Rating	Strong	Strong	Strong	Strong	N/A	N/A
Final Score	Reviewer discrepancy	No		No		No	
	Reason for discrepancy						
	**Final global rating**	**Strong**		**Strong**		**Moderate**	

		Zanoni et al., 2017	
	Team member	MD	HZ
Selection Bias	Represent population	Somewhat likely	Somewhat likely
	% agreed to participate	N/A	N/A
	Section Rating	Moderate	Moderate
Study Design	Study design	Retrospective cohort analysis	Retrospective cohort analysis
	Randomized	No	No
	Randomization described	No	No
	Randomization appropriate	No	No
	Section Rating	Moderate	Moderate
Confoun-ders	Difference between groups pre-intervention	Yes	Yes
	% of confounders controlled for	60-80%	60-79%
	Section Rating	Moderate	Moderate
Blinding	Assessors aware of intervention/exposure	N/A	N/A
	Participants aware of research question	N/A	N/A
	Section Rating	N/A	N/A
Data Collection	Data collection methods valid	Yes	Yes
	Data collection tools shown to be reliable	No	Yes
	Section Rating	Moderate	Moderate
Withdrawals and Drop-outs	Withdrawals/drop-outs reported in #s/reasons	N/A	N/A
	% completing the study	N/A	N/A
	Section Rating	N/A	N/A
Final Score	Reviewer discrepancy	No	
	Reason for discrepancy		
	**Final global rating**	**Strong**	
